# Anomalous changes in atmospheric radon concentration before and after the 2011 northern Wakayama Earthquake (Mj 5.5)

**DOI:** 10.1093/rpd/ncw142

**Published:** 2016-07-13

**Authors:** Mikako Goto, Yumi Yasuoka, Hiroyuki Nagahama, Jun Muto, Yasutaka Omori, Hayato Ihara, Takahiro Mukai

**Affiliations:** 1 Department of Biophysical Chemistry, Kobe Pharmaceutical University, 4-19-1 Motoyamakitamachi, Higashinada-ku, Kobe City, Hyogo 658-8558, Japan; 2 Institute of Radioisotope Research, Kobe Pharmaceutical University, 4-19-1 Motoyamakitamachi, Higashinada-ku, Kobe City, Hyogo 658-8558, Japan; 3 Department of Earth Science, Tohoku University, 6-3 Aza Aoba, Aramaki, Aoba-ku, Sendai City, Miyagi 980-8578, Japan; 4 Department of Radiation Physics and Chemistry, Fukushima Medical University, 1 Hikarigaoka, Fukushima City, Fukushima 960-1295, Japan; 5 Radioisotope Laboratory Center, Wakayama Medical University School of Medicine, 811-1 Kimiidera, Wakayama City, Wakayama 641-8509, Japan

## Abstract

A significant increase in atmospheric radon concentration was observed in the area around the epicentre before and after the occurrence of the shallow inland earthquake in the northern Wakayama Prefecture on 5 July 2011 (Mj 5.5, depth 7 km) in Japan. The seismic activity in the sampling site was evaluated to identify that this earthquake was the largest near the sampling site during the observation period. To determine whether this was an anomalous change, the atmospheric daily minimum radon concentration measured for a 13-year period was analysed. When the residual radon concentration values without the seasonal radon variation and the linear trend was > 3 standard deviations of the residual radon variation corresponding to the normal period, the values were deemed as anomalous. As a result, an anomalous increase in radon concentration was determined before and after the earthquake. In conclusion, anomalous change related to earthquakes with at least Mj 5.5 can be detected by monitoring atmospheric radon near the epicentre.

## Introduction

Radon (^222^Rn) concentration anomalies have been repeatedly correlated with seismic activity^(1–3)^. Radon concentration in soil is ~1000 times higher than that in the atmosphere. Radon generated from soil, rocks and ground water is released into soil pore air. Depending on the interconnected porosity, diffusion length, the presence of fractures and pressure gradient, radon can migrate to the Earth's surface. Groundwater and underground air are closely linked to the migration of radon in bedrock. In other words, radon present in voids relatively close to the Earth's surface (at depths where it does not appear at the Earth's surface under normal condition) move to the Earth's surface^(4)^.

As a noble gas, radon is chemically stable and can be detected with highly sensitive methods. In Japan, atmospheric radon concentrations have been estimated using ionisation currents recorded as exhaust monitoring measurements by some radioisotope (RI) institutes^(5–7)^. The exhaust monitors (airflow ionisation chamber with an effective volume of 1.4 × 10^−2^ to 1.8 × 10^−2^ m^3^) have ~10 times higher sensitivity than general continuous radon monitors such as AlphaGUARD (Saphymo GmbH, Frankfurt, Germany; a pulse-counting ionisation chamber with effective volume of 5.6 × 10^−4^ m^3^)^(7)^.

The measurements performed from January 1984 until February 1996 at the RI institute of Kobe Pharmaceutical University (N34.7°, E135.3°) recorded a significant increase in the atmospheric radon concentration from October 1994 until the 1995 Southern Hyogo Prefecture (Kobe) Earthquake (17 January 1995; Mj 7.3; depth 16 km; N34.6°, E135.0°; shallow inland earthquake)^(2, 3)^. Magnitude Mj is calculated only for large and shallow (depth ≤ 60 km) earthquakes using acceleration data of the seismic intensity metres at the Local Meteorological Observatories in Japan^(8)^. The temporal change in the atmospheric radon concentration was compared with the crustal strain, the groundwater radon concentration and the groundwater discharge rate prior to the earthquake. A previous study had reported that crustal strain fluctuations in the order of 10^−8^ could be linked to pre-seismic phenomena^(9)^. Therefore, our previous studies concluded that there is a correlation between atmospheric radon concentration and crustal strain in earthquakes of magnitudes smaller than the 1995 Kobe earthquake^(9)^. The present article aims to investigate the atmospheric radon concentration's variability before and after the shallow inland earthquake at the northern Wakayama Prefecture on 5 July 2011 (2011 northern Wakayama Earthquake Mj 5.5 (depth 7 km; N34.0°, E135.2°; hypocentral distance from the monitoring site: 22 km)).

**Figure 1.**
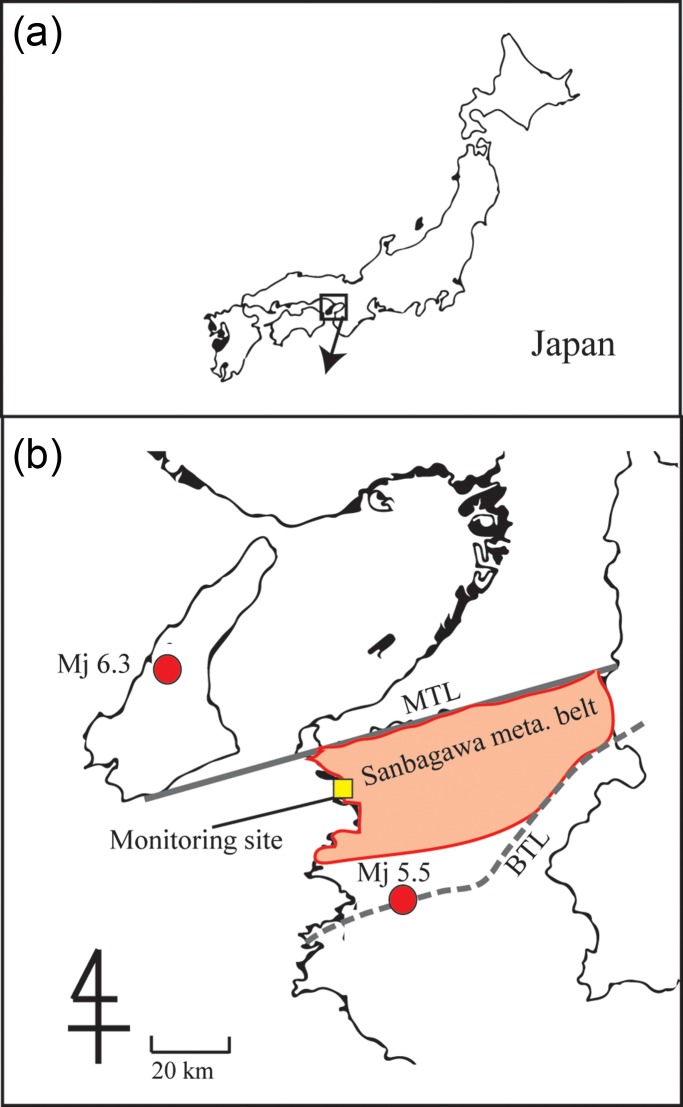
Location of the monitoring site, the corresponding epicentres and the tectonic lines. (**a**) Japan. (**b**) The Kii Peninsula. For interpretation of the references to colour in the text, the reader is referred to the electronic version of this article.

The radon data were obtained from one monitoring site (Figure 1) for a period of 13 years. These measurements covered two periods, the normal period (before the earthquake) and the validation period (during and after the earthquake). The residual radon concentration values after removing the annual components and the linear trend were analysed. When they were > 3 standard deviations (> ± 3σ) of the residual radon variation corresponding to the normal period, the values were deemed as anomalous. In addition, the seismic activity in the sampling site was evaluated to identify whether this earthquake was the largest near the sampling site during the observation period. Finally, we investigated whether there is a correlation between the observed radon anomalies and the seismic activity.

## Methods

### Monitoring site

The monitoring site is located at the Wakayama Medical University (N34.2°, E135.2°) in Wakayama City, Japan (Figure 1). It is placed within the Sanbagawa metamorphic belt and between the Median Tectonic Line (MTL) and the Butsuzo Tectonic Line (BTL)^(10)^. The area is the most seismically active region in the Kii Peninsula and is characterised by anomalously shallow swarm activity, crustal uplift and dilation^(11)^. The 2011 northern Wakayama Earthquake with magnitude Mj 5.5 occurred on 5 July 2011, and its epicentre (N34.0°, E135.2°) was located in the southern part of the area along the BTL^(12)^. The focal depth of the earthquake was 7 km and its hypocentral distance from the monitoring site was 22 km. The 2013 Awaji Island Earthquake Mj 6.3 (depth 15 km; N34.4°, E134.8°; hypocentral distance from the monitoring site: 44 km) also occurred on 13 April 2013.

### Radon concentration measurements

**Figure 2.**
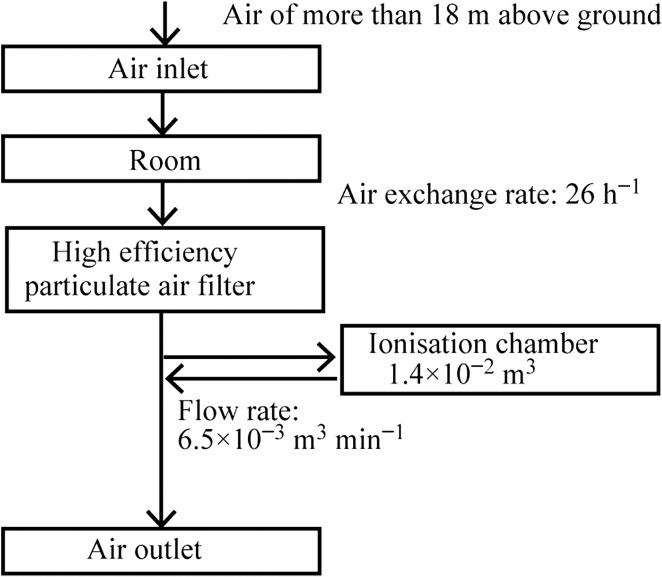
Schematic block diagrams showing the radon measurement system.

A gas-flow ionisation chamber (Hitachi, Ltd., Tokyo, Japan; model: DGM-101; effective volume: 1.4 × 10^−2^ m^3^; flow rate: 6.5 × 10^−3^ m^3^ min^−1^; equipped with ^90^Sr calibration source) was used at the monitoring site to monitor RI leakage in exhaust air from the RI institute (Figure 2). Air from ~18 m above the ground was brought in the RI institute at an air-exchange rate of 26 h^−1^ through forced draught fans. The ventilation system was operated on a 24 h basis. The exhaust air passed through a high-efficiency particulate air filter to prevent RI leakage. In addition, this filter removed radon progenies. Then, part of the exhaust air was led into the ionisation chamber. The hourly atmospheric radon concentration in the gas-flow ionisation chamber was continuously measured. Following Tajika *et al*.^(7)^, the data set was converted from ionisation current to radon concentration values (conversion factor 1.8 Bq m^−3^ fA^−1^).

The 2011 northern Wakayama Earthquake occurred during the observation period from 1 January 2000 to 21 June 2013. The normal period was determined to be 1 January 2000 to 31 December 2010, and the validation period included the interval from 1 January 2011 to 21 June 2013. The data on the 29th of February in the leap years were excluded. Missing data (<1%) were linearly interpolated.

In this article, we investigate the variation in radon concentration before and after the earthquake. For the atmospheric radon level, the gas-flow ionisation chamber can detect the atmospheric radon with high precision. However, knowledge of the background atmospheric radon level is necessary to determine the absolute radon concentration values accurately. Therefore, absolute values of radon concentration were not determined in this article.

A previous study provided evidence that variations in outdoor radon concentration could be determined using gas-flow ionisation chambers for RI monitoring systems^(7)^, when air flowed through forced draught fans at an exchange rate of 4.9 h^–1^. Therefore, we considered that an air-exchange rate of 26 h^–1^ was enough to measure atmospheric radon. In addition, we confirmed that the effects of RIs used in this RI institute can be ignored.

The validation period included the nuclear accident at the Fukushima Daiichi nuclear power station on 11 March 2011. Because of the accident, there was significant long-range transport of gaseous ^131^I and other radionuclides from Fukushima. However, the monitoring site (N34.2°, E135.2°) was located in the west part of Japan and >600 km west–southwest of the Fukushima Daiichi nuclear power station (N37.4°, E141.0°). Air mass is generally carried by the wind to the east around Japan. Therefore, the monitoring site was located out of the transport area of gaseous ^131^I^(13)^. Consequently, we disregarded the effect of gaseous ^131^I.

## Data analysis

**Figure 3.**
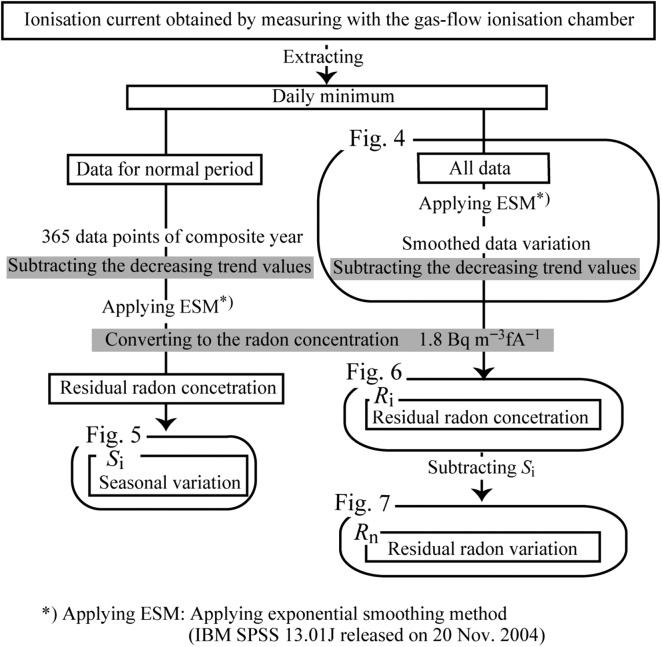
Schematic block diagrams showing the analysis and calculation methods.

**Figure 4.**
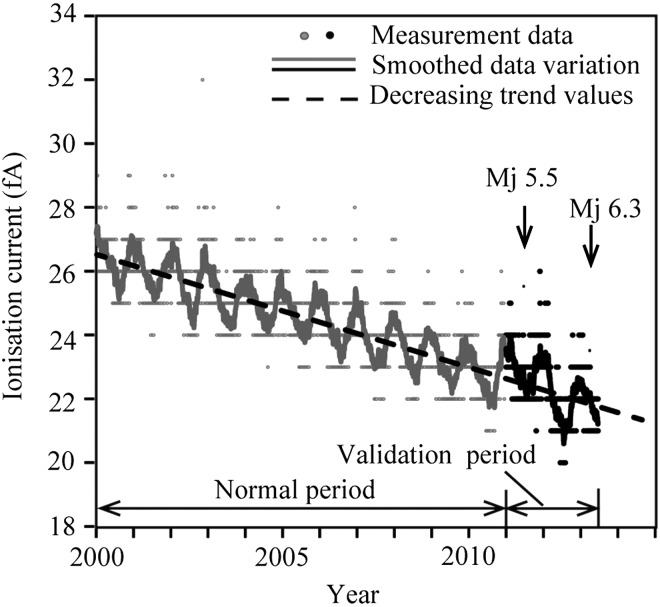
Time series of the ionisation current data variation and the decreasing trend values.

The daily minimum radon concentration can be used when examining seismic anomalous radon variations^(9)^. Figure 3 shows a block diagram of the data analytical process. In the present study, the atmospheric radon concentration measured in the afternoon was considered as the daily minimum value because the radon concentration in well-mixed air is insensitive to changes in the mechanical mixing strength (e.g. wind speed). The daily minimum data, which were extracted from the ionisation current measurements with the ionisation chamber, showed seasonal variability and an overall decreasing trend (Figure 4). The seasonal variations in the radon concentration in air were attributed to changes in the atmospheric conditions such as temperature. The decreasing trend was considered to be due to the decay of equipment ^90^Sr with the ionisation chamber. The daily minimum data were divided into two periods: the normal period (from 1 January 2000 to 31 December 2010) and the validation period (1 January 2011 to 21 June 2013). The seasonal variation and decreasing trend were calculated using the data for the normal period as shown below.

**Figure 5.**
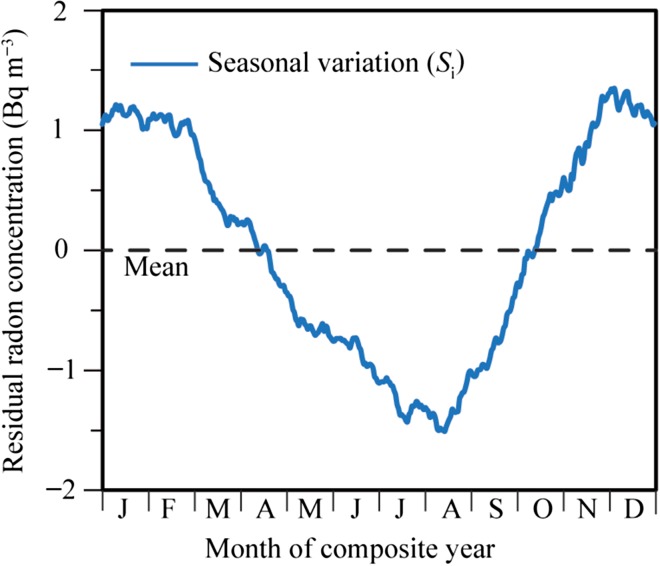
Seasonal variation (*S*_i_) in the residual radon concentration. This figure is modified from Kobayashi *et al*.^(14)^.

Next, using daily minimum values of the measured ionisation current data during the normal period for calculating the seasonal variation, the average data for each day of the year during all the years of the normal period (365 data points of composite year in Figure 3) were calculated. The decreasing trend values (in Figure 4; described below in more detail) were subtracted from the average data, and the exponential smoothing method (IBM SPSS 13.01 J released on 20 November 2004) was applied. Then, the ionisation current data were converted to the radon concentration. The normal seasonal variation in the daily minimum radon concentration (residual radon concentration) *S*_i_ (Figure 5) was obtained. Our previous studies reported that the seasonal variation in Figure 5 was the typical seasonal variation in Japan^(14, 15)^, and approximated the inverse correlation of the annual variation pattern for the surface temperature. Thus, the seasonal variation in Figure 5 could be applied as the typical seasonal variation for the seasonal variation in Figure 4.

**Figure 6.**
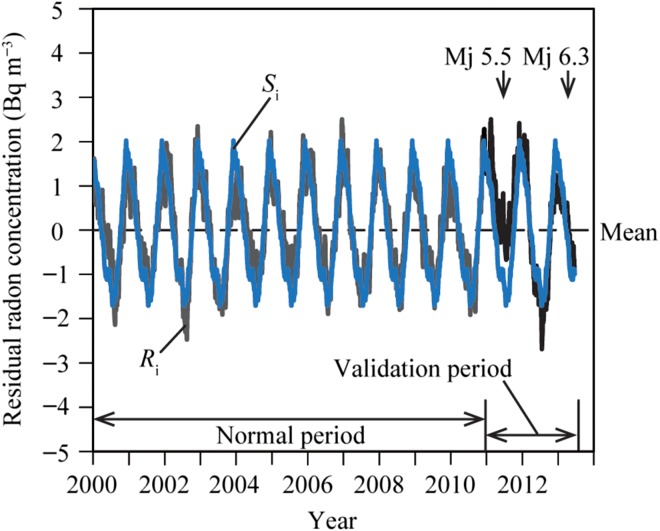
Time series of the residual radon concentration variations. Comparison between the time series of the residual radon concentration variation (*R*_i_: grey and black lines) and the seasonal variation (*S*_i_: blue line). For interpretation of the references to colour in the text, the reader is referred to the electronic version of this article.

**Figure 7.**
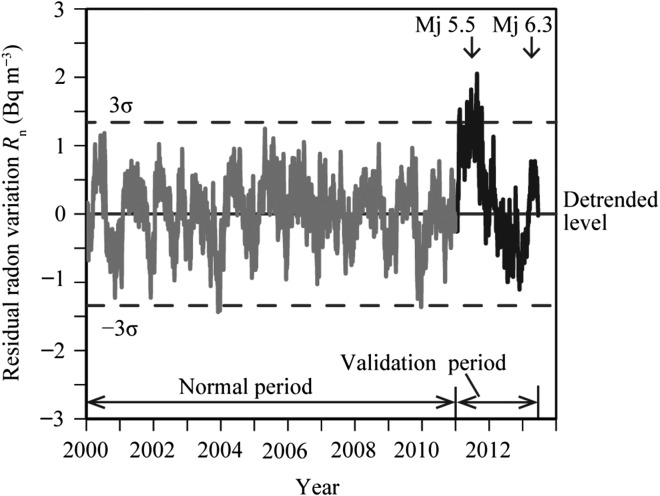
Time series of the residual variations in radon concentration from the detrended level. The broken line indicates ± 3σ obtained for the normal period.

Next, using the daily minimum values of the measured ionisation current data during the entire observation period, the time series of the residual radon variation (*R*_n_) from the detrended level was calculated on the detrended data (Figure 3)^(15)^. Applying the exponential smoothing method to the variation in the daily minimum data, the smoothed data variation was obtained in Figure 4. The smoothed data variation was divided into the variation during the normal period (grey line) and the variation during the validation period (black line). The decreasing trend values caused by the decay of equipment ^90^Sr shown in Figure 4 were subtracted from the smoothed data values. By converting the ionisation current data to the radon concentration, the residual radon concentration (*R*_i_) was obtained in Figure 6. The time series of the residual radon variation *R*_n_ was calculated by subtracting *S*_i_ from *R*_i_ in Figure 7.

## Results and Discussion

Figure 6 shows the time series of *R*_i_ and *S*_i_, where *R*_i_ is divided into the variation during the normal period (grey line) and the variation during the validation period (black line). The anomalous increase in the radon concentration is determined by the values > ± 3σ of the residual radon variation during the normal period. Figure 7 presents the variation in *R*_n_ during the entire observation period and the ± 3σ lines, the grey line of *R*_n_ indicates the variation during the normal period, and the black line of *R*_n_ indicates the variation during the validation period. Anomalous increases in the radon concentration > ± 3σ are observed before and after the 2011 northern Wakayama Earthquake (Mj 5.5), which occurred on 5 July 2011.

**Figure 8.**
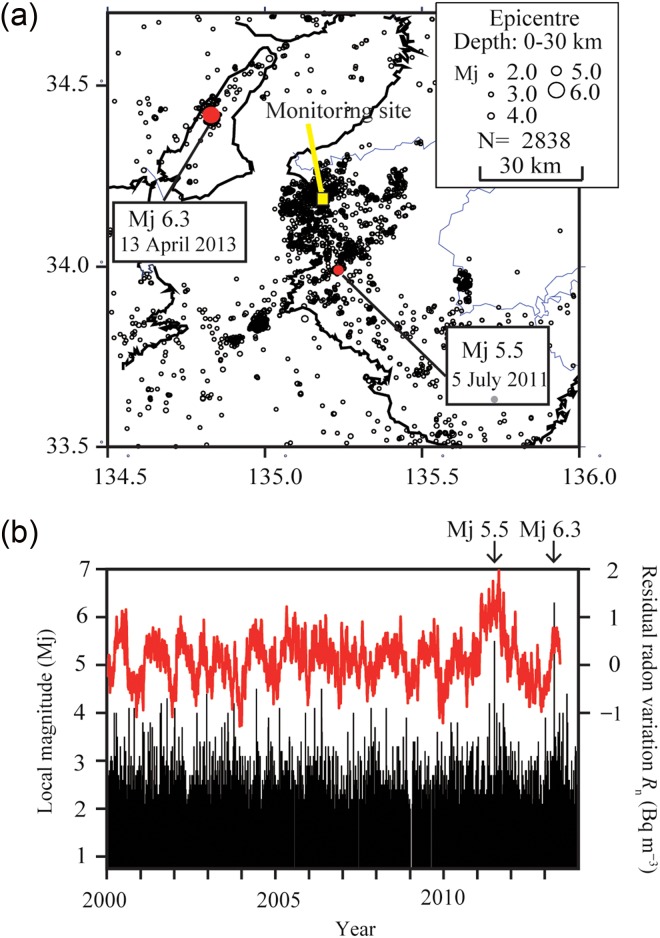
Earthquake activity occurred the same period as the observation of atmospheric radon around the monitoring site. (**a**) The distributions of earthquake epicentres around the monitoring site (from January 2000 to December 2013). (**b**) Magnitude-time diagram of earthquakes (black line represented by left vertical axis) in the area shown in panel a and the time series of the residual variations (red line represented by right vertical axis) shown in Figure 7. Data were provided by Japan Meteorological Agency through the website^(16)^. For interpretation of the references to colour in the text, the reader is referred to the electronic version of this article.

Figure 8a shows the distributions of earthquakes around the monitoring site for the same period as the observation of atmospheric radon (from January 2000 to December 2013)^(16)^. Figure 8b shows a magnitude-time diagram for the same area in the same period^(16)^. As shown in Figure 8, during the 13-year observational period, there is only one earthquake larger than Mj  >  5.0 (2013 Awaji Island Earthquake) in addition to 2011 Wakayama Earthquake.

We discuss the relationship between radon changes and the 2013 Awaji earthquake, and other events occurred outside of the region of Figure 8. The *R*_n_ values before and after the 2013 Awaji Island Earthquake (Mj 6.3) were within the normal variation range (Figures 7 and 8). Moreover, the *R*_n_ before and after earthquakes (Mj  ≥  5.5) shown in Table 1, which occurred around the monitoring site (hypocentral distance lower than 220 km), except the 2011 northern Wakayama Earthquake, also remains within the normal variation range. Earthquakes shown in Table 1 were collected in accordance with the above criteria and with reference to the same earthquake prediction research^(16)^, which were proposed for radon variation in soil and ground water^(17–20)^.

**Table 1.** Earthquakes (Mj ≥  5.5) occurred around the monitoring site (hypocentral distance < 220 km)^(17)^.

Occurrence date (earthquake name)	Location	Mj^(8)^	Depth (km)	Hypocentral distance^a^ (km)
6 October 2000	N35.3°, E133.3°	7.3	9	207
8 October 2000	N35.1°, E133.2°	5.6	7	214
31 October 2000	N34.3°, E136.3°	5.7	39	113
12 January 2001	N35.5°, E134.5°	5.6	11	155
16 September 2002	N35.4°, E133.7°	5.5	10	186
5 September 2004	N33.1°, E137.1°	7.4	44	220
5 July 2011 (2011 northern Wakayama Earthquake)	N34.0°, E135.2°	5.5	7	22
13 April 2013 (2013 Awaji Island Earthquake)	N34.4°, E134.8°	6.3	15	44

^a^Hypocentral distance from the monitoring site.

Anomalous variations in the daily minimum atmospheric radon concentration were recorded at the Wakayama Medical University from 9 February 2011 to 3 September 2011. The period of the anomalous variations includes the 2011 northern Wakayama Earthquake. This phenomenon might be an indication of changes in the radon concentration, due to exhalation by the seismic anomaly around the monitoring site, where the active earthquake swarm occurred.

## Conclusions

An anomalous change related to earthquakes with at least Mj 5.5 can be detected by monitoring atmospheric radon near the epicentre. These anomalies are higher than ± 3σ, and are calculated using the normal period data. It was supported by our demonstrations that the temporal change in the atmospheric radon concentration retain a potential to occur with that in the crustal strain of not only a shallow inland earthquake of Mj 7.3 (1995 Kobe earthquake) but also a shallow inland earthquake of the smaller magnitude. A seismic radon response is anticipated for Mj 5.5 level earthquakes. Therefore, the present study reveals the importance of monitoring atmospheric radon in order to investigate the possible earthquake mechanisms.
